# Influence of p53 Isoform Expression on Survival in High-Grade Serous Ovarian Cancers

**DOI:** 10.1038/s41598-019-41706-z

**Published:** 2019-03-27

**Authors:** Katharina Bischof, Stian Knappskog, Sigrun M. Hjelle, Ingunn Stefansson, Kathrine Woie, Helga B. Salvesen, Bjorn T. Gjertsen, Line Bjorge

**Affiliations:** 1 Centre for Cancer Biomarkers CCBIO, Department of Clinical Science, University of Bergen, 5020 Bergen, Norway; 2 Department of Gynecology and Obstetrics, Haukeland University Hospital, 5021 Bergen, Norway; 3 Department of Oncology, Haukeland University Hospital, 5021 Bergen, Norway; 4 Section of Oncology, Department of Clinical Science, University of Bergen, 5020 Bergen, Norway; 5 Department of Pathology, Haukeland University Hospital, 5021 Bergen, Norway; 6 Centre for Cancer Biomarkers CCBIO, Department of Clinical Medicine, Section for Pathology, University of Bergen, 5020 Bergen, Norway; 7 Department of Internal Medicine, Haematology Section, Haukeland University Hospital, 5021 Bergen, Norway

## Abstract

High-grade serous ovarian carcinoma (HGSOC) is characterised by alterations in the p53 pathway. The expression levels of p53 isoforms have been shown to be associated with patient survival in several cancers. This study examined the predictive and prognostic effects of the expression levels of *TP53* pre-mRNA splicing isoforms and *TP53* mutations in tumour tissues in 40 chemotherapy responders and 29 non-responders with HGSOC. The mRNA expression levels from total p53, and total Δ133p53, p53β, p53γ isoforms were determined by RT-qPCR, and *TP53* mutation status by targeted massive parallel sequencing. The results from these analyses were correlated with the clinical outcome parameters. No differential expression of p53 isoforms could be detected between the chemosensitive and chemoresistant subgroups. In a multivariate Cox regression model, high levels of total Δ133p53 were found to be an independent prognosticator for improved overall survival (HR = 0.422, p = 0.018, 95% CI: 0.207–0.861) and reached borderline significance for progression-free survival (HR = 0.569, p = 0.061, 95% CI: 0.315–1.027). *TP53* mutations resulting in loss of function or located at known hotspots were predictive of tumour characteristics and disease progression. These findings suggest that total Δ133p53 mRNA can be a biomarker for survival in HGSOC.

## Introduction

Mutations in the *TP53* gene are early and almost ubiquitous events in the genesis of high-grade serous ovarian carcinoma (HGSOC)^1–4^. Although various classes of mutations occur throughout the coding region of the *TP53* gene in human cancers, there is a clear enrichment of missense or nonsense single base substitutions affecting the DNA-binding domain of the protein^5^. Since large-scale sequencing data for *TP53* mutations became available, the predictive and prognostic roles of defined subcategories of *TP53* mutations have been extensively investigated. Alterations that result in a loss of function (LOF) or oncogenic mutations conferring to tumour-promoting abilities have been shown to be associated with chemoresistance and worse survival in ovarian cancers^6–9^. Small molecule therapies targeting mutated p53 proteins in cancer cells are under development for the treatment of ovarian cancer and may lead to *TP53* mutational guided therapy^10,11^.

**Figure 1:**
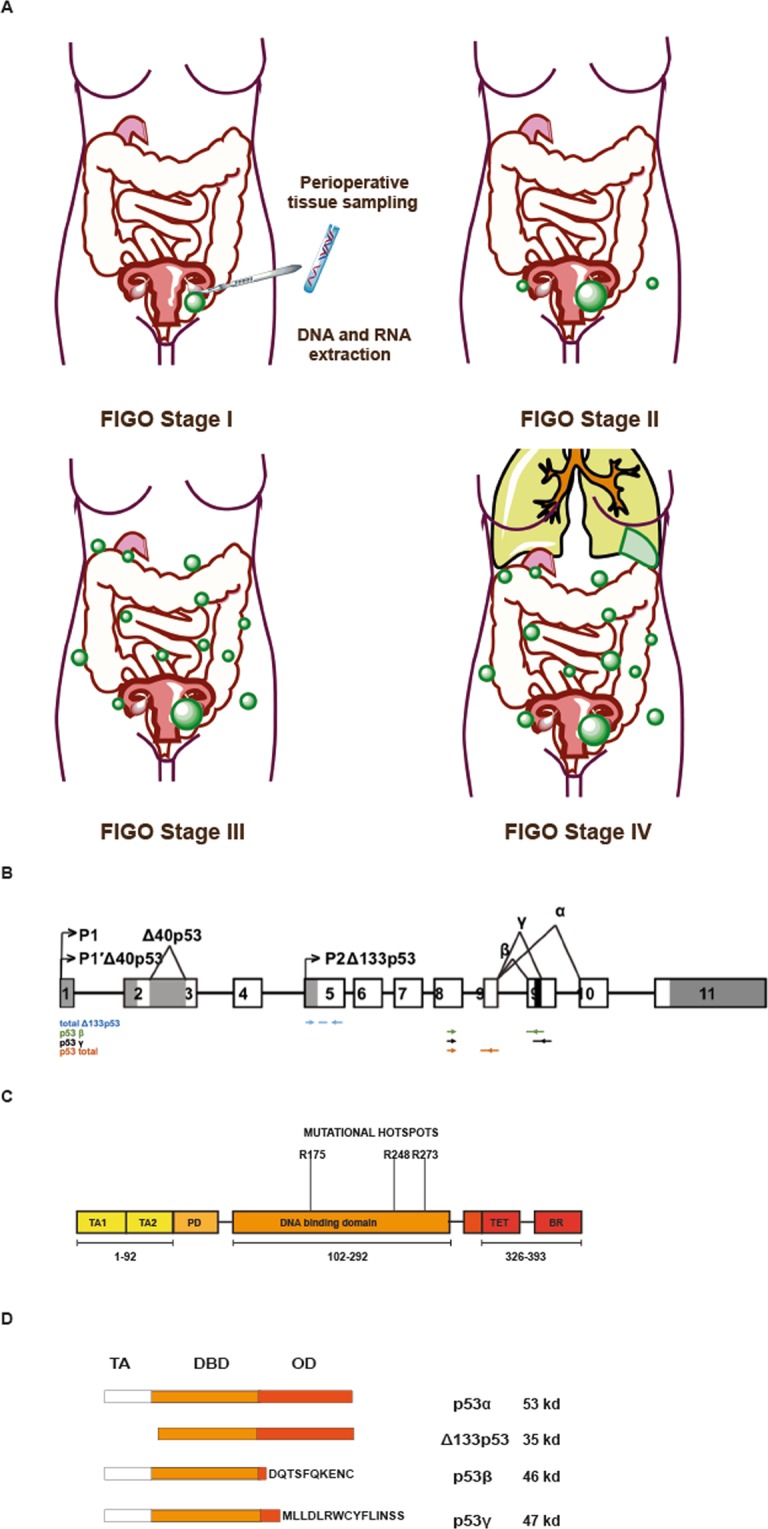
(**A**) Overview of FIGO disease stages represented by the series for this study and illustration of sample collection and extraction of DNA and RNA. (**B**) Structure of the human TP53 gene comprising 11 exons. P1 = proximal promoter encoding full-length p53, P2 = internal promoter resulting in Δ133p53 product. Alternative splicing sites (^). Primer location is indicated by coloured arrows and is indexed on the lower left. (**C**) Provides a more detailed schematic illustration of the distinct functional and structural domains of the 393 amino acid long p53 protein. TA1 and TA2 form the transactivation domain, followed by the proline rich domain (PD) and the DNA binding domain, where the three most frequently found point mutations in high-grade serous gynaecological cancers are indicated. The tetramerization domain (TET) and basic region (BR) form the C-terminus. (**D**) Illustrates the exon composition of canonical p53 and relevant p53 isoforms. Abbreviations: Transactivation domain (TA), DNA binding domain (DBD), C-terminal oligomerization domain (OD). The N-terminally truncated isoform Δ133p53 is a product of the regulation of an internal promoter in intron 4 (P2). The C-terminally altered p53β and p53γ isoforms have alternative sequences after amino acid 332. Molecular weight indicated in kilodalton (kd) on the right side.

In humans, the *TP53* gene encodes RNA that is edited by pre-mRNA splicing, yielding at least 12 protein isoforms expressed to various degrees in different tissues and under various physiological conditions^12^ (Fig. 1). Emerging evidence has linked the deregulated expression of these isoforms to cancer^13–18^. The amino-terminally truncated Δ133p53 isoform is the product of alternative promoter usage and has been shown to exert dominant-negative functions toward canonical p53 in colon cancer cell line models^18^. In addition, Δ133p53 has been shown to inhibit replicative senescence and to promote cellular proliferation^13^. The biological role of the carboxy-terminally truncated p53β and p53γ isoforms, both products of alternative splicing of intron 9^18–20^, is still being debated. However, these isoforms have been shown to enhance p53´s tumour-suppressive transcriptional activity of Bax and p21^21,22^.

Previous studies, including those by the authors, have reported that in gynaecological malignancies, such as epithelial ovarian cancers and uterine serous cancers, the altered expression of the p53 isoforms was associated with tumour traits, such as grade of differentiation, sensitivity to chemotherapy and survival^23–26^.

Chemotherapy resistance remains a major obstacle in the successful treatment of patients with HGSOC. Data from high-resolution copy-number profiles on tumour samples collected throughout therapy indicate that HGSOC already exhibits substantial clonal diversity at primary diagnosis^27^; therefore, the high rates of acquired chemoresistance may be a result of subset selection with primary innate chemoresistance^28^.

In the current study, we aimed to investigate the predictive and prognostic impact of the p53 isoforms in a cohort of HGSOC patients selected based on their response or non-response to chemotherapy. The potential predictive and prognostic roles were explored based on the mRNA expression levels of the individual *TP53* isoforms in HGSOC tumour tissue (Fig. 1C). High mRNA levels of the amino-terminally altered total Δ133p53 isoform were associated with favourable survival. These findings suggest the total Δ133p53 isoform to be a possible biomarker for disease progression in HGSOC, worthy of further validation.

## Results

### Prognostic value of clinical characteristics

The sample in the present study comprised 69 patients with HGSOC. These 69 cases were selected from a cohort of 493 epithelial ovarian cancers (EOCs) (see Methods section) based on their poor (n = 29) responses or good (n = 40) responses to adjuvant treatment with carboplatin and taxane, thus representing chemoresistant and chemosensitive subsets, respectively. A poor response to adjuvant therapy was defined as disease progression after ≤6 months after completed primary platinum/taxane based chemotherapy, and a good response was ≥17 months of progression-free survival (PFS). The mean age at the time of diagnosis was 59 (range 31–75) for poor responders and 61 (range 44–77) for good responders. It was noted that 72% (21 of 29) of the poor responders presented with International Federation of Gynecology and Obstetrics (FIGO) stage IIIC disease at the time of primary treatment, and 90% (36 of 40) of good responders were diagnosed at stage IIIC. In both cohorts, a majority of the tumours exhibited high-grade differentiation (see Table 1.)

**Table 1:** Distribution of clinicopathologic characteristics for 69 patients within subgroups of high grade FIGO stages ≥ IIIC enriched for either good response; ≥17 months progression free survival after completed primary treatment (surgery followed by adjuvant chemotherapy) or poor response with disease progression within 6 months after completed primary treatment.

Clinical parameters	Poor response	Good response
	Mean (range)	Mean (range)
Age at diagnosis; in years	59 (31–75)	61 (44–77)
Time to progression; in months	3 (1–6)	42 (17–137)
	N (%)	N (%)
Tumor grade		
Grade 2	8 (28%)	7 (17%)
Grade 3	21 (72%)	33 (83%)
FIGO stage		
IIIC	21 (72%)	36 (90%)
IV	8 (28%)	4 (10%)
Macroscopic surgical resection (data missing for 5 respectively 8 cases)		
Complete	—	16 (50%)
Residual disease ≤1 cm	16 (67%)	12 (38%)
Residual disease >1 cm	8 (33%)	4 (12%)

Univariate and multivariate Cox regression analyses (see Table 2) were performed to determine the prognostic relevance of the clinicopathological prognosticators in the combined sample set (n = 69), and disease stage was found to be a predictor of PFS (univariate analysis [p = 0.007]; multivariate analysis [hazard ratio = 2.317, p = 0.033, 95% CI: 1.071–5.012]). The results of the univariate analysis showed disease stage to be significantly associated with overall survival (OS), (p = 0.002), and the results of the multivariate model showed borderline significance (hazard ratio = 2.299, p = 0.051, 95% CI: 0.998–5.297). The presence of residual disease after surgery also had an effect on PFS, as seen in the univariate risk model (p = 0.032), and OS, as seen in the univariate analysis (p = 0.017) and the multivariate analysis (hazard ratio = 3.735, p = 0.041, 95% CI: 1.054–13.240). Other clinicopathological variables, such as patient age and tumour grade, had no significant effect on the survival parameters.

**Table 2:** Established, clinical prognosticators for survival in univariate and multivariate Cox regression analysis in 69 patients with HGSOC. Abbrevations: HR = Hazard ratio. CI = Confidence interval.

	Progression-free survival Multivariate			Overall survival Multivariate		
	Univariate P-value	HR (95% CI)	P-value	Univariate P-value	HR (95% CI)	P-value
Age at diagnosis; in years	0.557	0.994 (0.958–1.032)	0.77	0.257	0.976 (0.934–1.021)	0.292
Disease stage III vs. IV	0.007	2.317 (1.071–5.012)	0.033	0.002	2.299 (0.998–5.297)	0.051
Tumor grade II vs. III	0.65	0.843 (0.411–1.731)	0.643	0.956	1.135 (0.456–2.826)	0.785
Residual disease 0 vs >0	0.032	1.810 (0.830–3.950)	0.136	0.017	3.735 (1.054–13.240)	0.041

### mRNA levels of p53 isoforms in HGSOC

**Figure 2:**
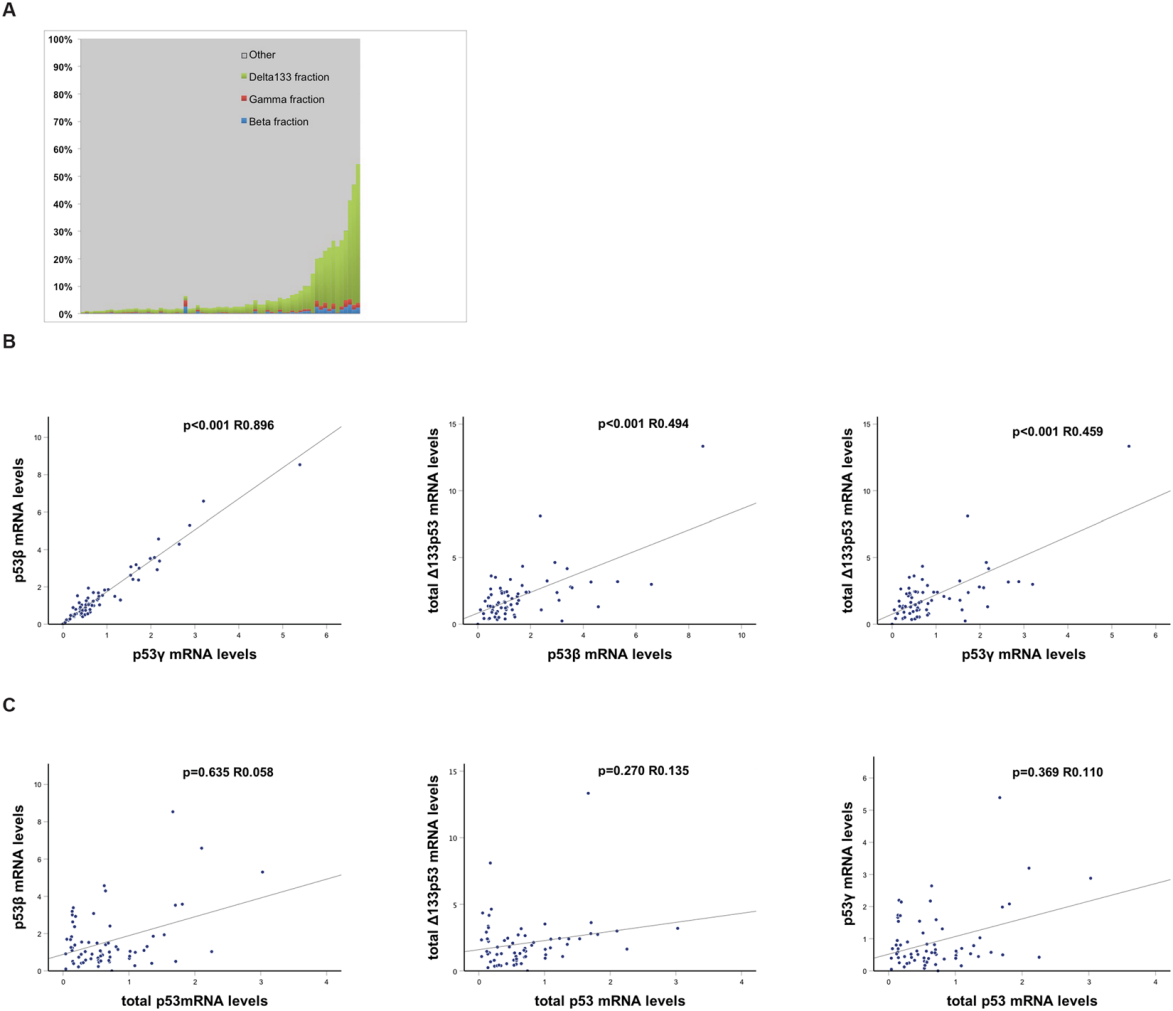
mRNA expression of p53 isoforms in tumour samples illustrated as (**A**). Histogram displaying fractions of p53 isoforms to total p53 mRNA in individual specimens. (**B**) Expression of p53 isoforms between each other; p53γ versus p53β, p53β versus Δ133p53, p53γ versus Δ133p53. (**C**) Expression levels of total p53, together with individual p53β, Δ133p53 and p53γ isoforms.

The levels of the total p53 mRNA were quantified, and breakpoint-specific qPCR assays were applied to detect the amino-terminal truncated total Δ40p53 and total Δ133p53 isoforms, and the carboxy-terminal truncated p53β and p53γ isoforms. Thus, the levels of the p53 transcripts containing each of the breakpoints, but not the combined mRNA species (e.g. Δ133p53β and Δ133p53γ) could be quantified. We identified the p53 isoforms total Δ133p53 and the splice isoforms p53β and p53γ in all but one of the samples (68 of 69); however, the expression levels of the total Δ40p53 mRNA could not be traced in any of the analysed samples. The landscape of the p53 isoforms was dominated by total ∆133p53 isoforms, which represented up to 50.6% of the total p53 mRNA; p53β and p53γ accounted for a maximum of 3.3% and 2.5% of the total pool of p53 isoforms, respectively (Fig. 2A) The p53γ isoform showed the largest variability between the lowest and the highest expressing samples (108-fold). The differences between the highest and the lowest expressing samples for the p53β and total ∆133p53 isoforms were 86-fold and 54-fold, respectively.

### Correlation between levels of p53 isoforms and total p53

The mRNA levels of the total Δ133p53 isoforms and the p53β and p53γ isoforms were all highly significantly correlated (p53β vs. p53γ R = 0.896, p < 0.001; p53β vs. total Δ133p53 R = 0.494, p < 0.001; p53γ vs. total Δ133p53 γ R = 0.459, p < 0.001; Fig. 2B). The expression levels of the individual isoforms were found to be independent of the total p53 levels (p > 0.25 for all of the comparisons; Fig. 2C).

### Relative expression of p53 isoforms to total p53 associated with *TP53* mutation status

**Figure 3:**
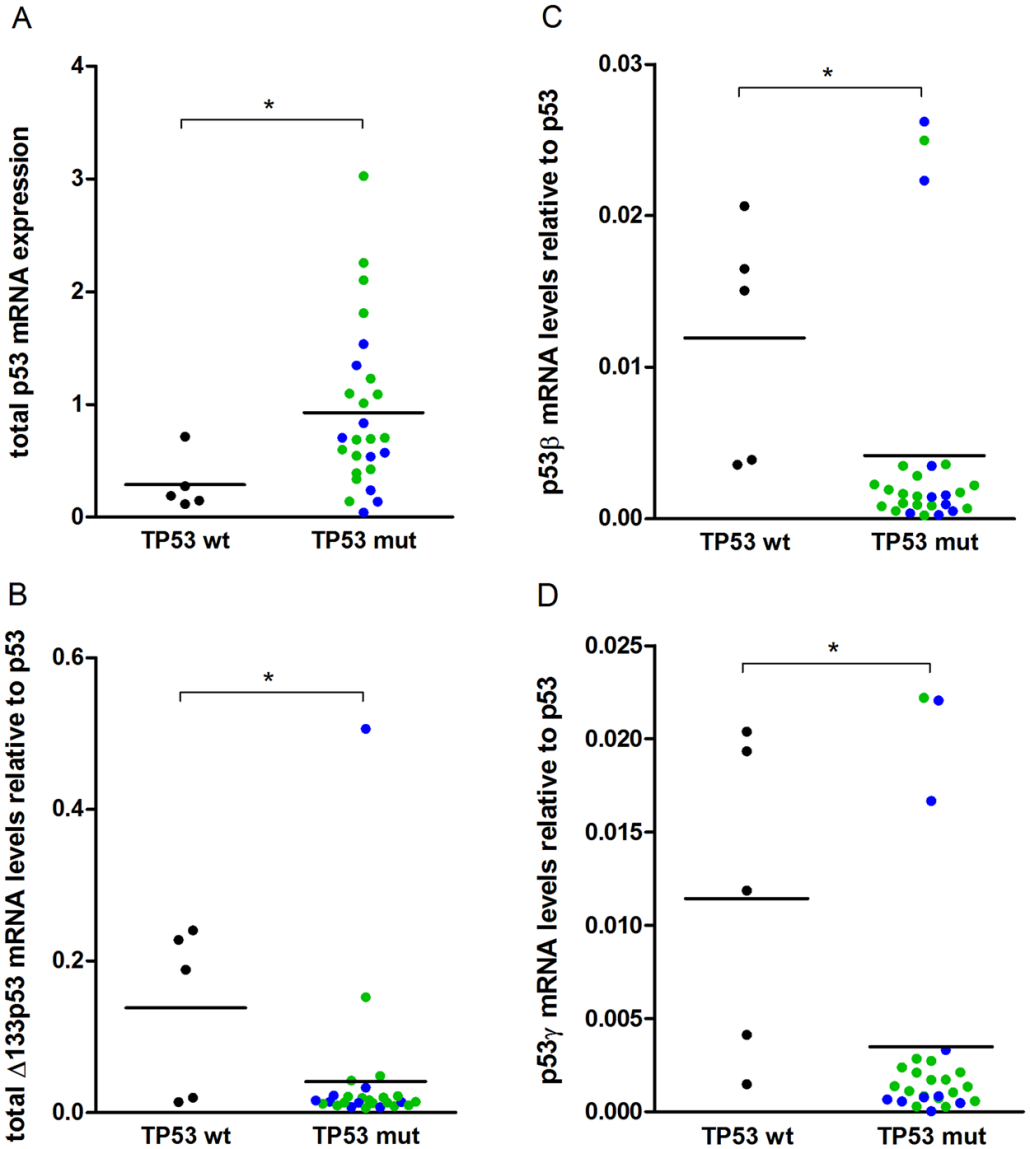
mRNA expression levels in individual tumour samples stratified for *TP53* mutation status for (**A**). Total p53 mRNA, (**B**) p53β mRNA relative to total p53, (**C**) Δ133p53 mRNA relative to total p53, (**D**) p53γ mRNA relative to total p53. *Denotes significance level p ≤ 0.05 blue colour indicates unclassified *TP53* mutation, green colour denominates the LOF/hot spot *TP53* mutation category.

The levels of total p53 were significantly lower in the *TP53* wild-type tumours (n = 5) than in the mutated specimens (n = 26; p = 0.036) (Fig. 3A). While the absolute mRNA levels of the p53 isoforms were not correlated with the *TP53* mutation status, the relative expression levels of the p53 isoforms as a fraction of the total p53 levels were higher in the wild-type specimens compared to the mutated specimens (total Δ133p53 relative to total p53 [p = 0.036], p53β relative to total p53 [p = 0.006], p53γ relative to total p53 [p = 0.016]; Fig. 3B–D).

### Prognostic effect of p53 isoforms

**Figure 4:**
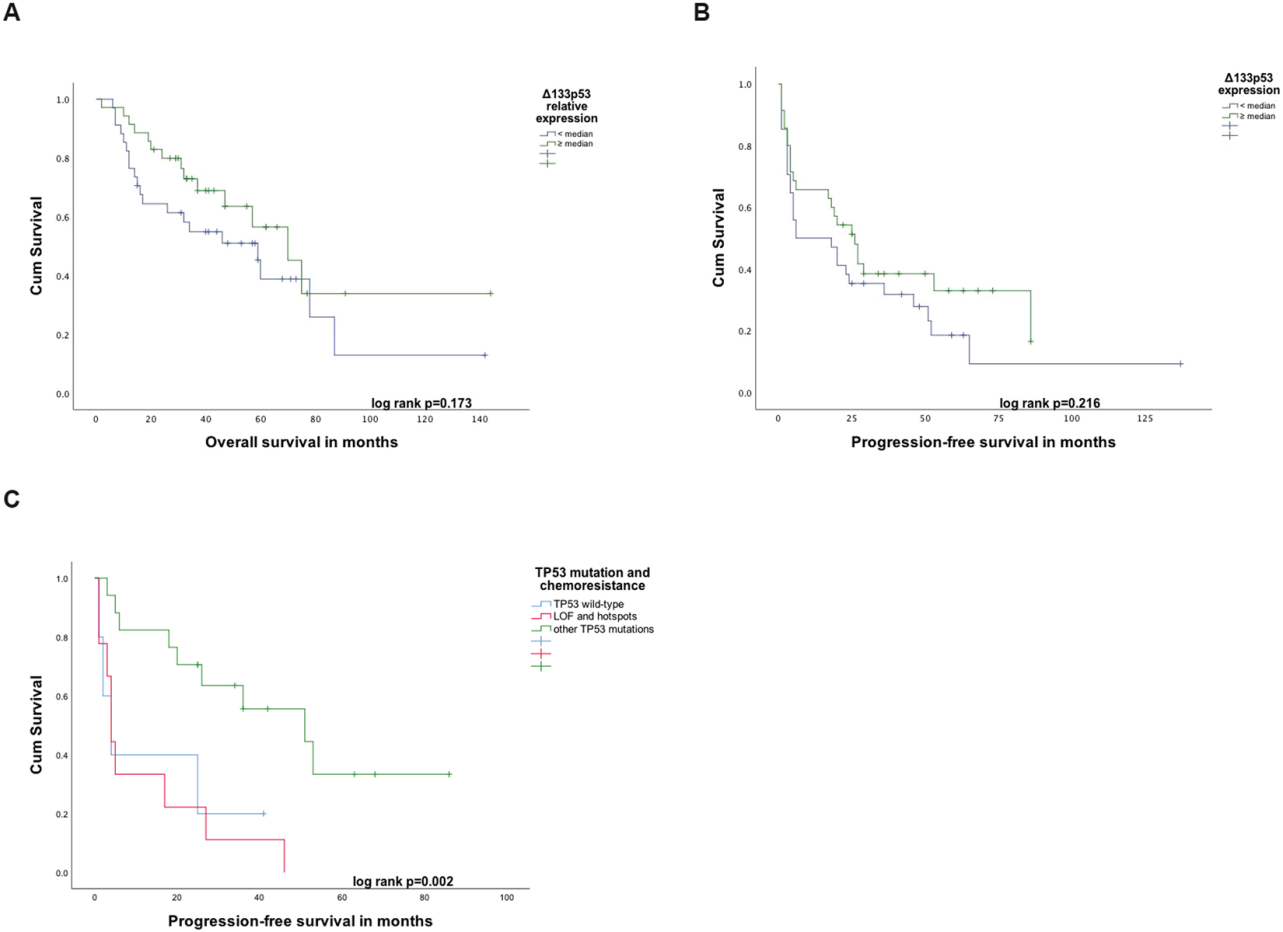
Kaplan-Meier survival plots for differential survival in patients expressing. (**A**) Higher vs. lower than median levels of Δ133p53 relative to total p53. (**B**) Illustration of PFS in patients, divided by median expression of Δ133p53 mRNA. (**C**) Kaplan-Meier survival plot for patients carrying cancers classified as *TP53* wild-type, LOF and hotspot mutations vs. other *TP53* mutations.

In further analyses, the levels of each isoform and the relative level, as a fraction of the total p53 levels, were used for comparisons with the clinical parameters. In the univariate OS analysis, the relative expression of total Δ133p53 was weakly associated with the prognosis (logrank p = 0.173; Fig. 4A). This association was further examined in a Cox proportional-hazards survival model. Known clinical prognostic factors for ovarian cancer patients (age at diagnosis grouped by median, tumour stage and presence of residual tumour) were introduced as binary variables, together with the relative Δ133p53 expression grouped by median (Δ133p53ratio high vs. low). The relative expression of total Δ133p53 isoforms to total p53 was found to be associated with longer OS (hazard ratio = 0.422, p = 0.018, 95% CI: 0.207–0.861 Table 3). For the other isoforms, no prognostic role could be established in the univariate analyses (Fig. S1) or the multivariate analyses.

**Table 3:** Prognostic and predictive impact of the p53 splice variant Δ133p53 for 69 women with HGSOC in multivariate Cox regression analysis.

	Progression free survival		Overall survival	
	HR (95% CI)	P-value	HR (95% CI)	P-value
Age at diagnosis; in years	0.993 (0.956–1.031)	0.710	0.975 (0.931–1.021)	0.274
Tumor stage IIIC vs. IV	2.694 (1.259–5.765)	0.011	2.357 (1.092–5.088)	0.029
Residual disease; in cm 0 vs. >0	1.928 (0.911–4.077)	0.086	5.254 (1.481–18.642)	0.010
Δ133p53 High vs. Low	0.569 (0.315–1.027)	0.061		
High vs. Low			0.422 (0.207–0.861)	0.018

Δ133p53ratio denominates mRNA expression of Δ133p53 as a fraction of the total p53 levels.

### p53 isoforms as predictive marker for sensitivity to chemotherapy

No significant difference was observed in the expression levels of the isoforms between the two predefined groups of chemoresistant and chemosensitive patients (Δ133p53 p = 0.135; p53β p = 0.961; p53γ p = 0.496; Mann-Whitney rank test). PFS was regarded as a surrogate marker for the efficacy of first-line chemotherapy and chemosensitivity. Higher than median levels of Δ133p53 were not associated with significantly altered time to progression, based on the results of the univariate survival analysis using the Kaplan-Meier estimator (logrank p = 0.216; see Fig. 4B). In the multivariate survival analyses, the expression of Δ133p53 grouped by median (Δ133p53 high vs. low) reached borderline significance for PFS (hazard ratio = 0.569, p = 0.061, 95% CI: 0.315–1.027 Table 3). For the other isoforms, the results of the univariate analyses (Fig. S2) and the multivariate analyses showed no association between the absolute or relative expression and survival.

### Associations between p53 isoform levels and clinicopathological parameters

No significant differences in the p53 isoform expression were observed between the completely resected patients and the patients for whom complete resection could not be achieved. In addition, a comparison of the moderate and low differentiated cases showed no differences in the p53 isoform levels. The expression of the p53γ isoform was associated with age when grouped by median (p = 0.044); however, the expression of the other isoforms showed no association with patient age. The levels of the p53β isoform relative to the total p53 were significantly higher expressed in the FIGO stage III vs. IV (p = 0.05) specimens.

### Clinical relevance of *TP53* mutation status

The combined data for the *TP53* mutation status and the p53 isoform expression were available for 31 patients. The main reason for this limited number of cases was lack of access to germline DNA (needed to distinguish somatic and germline variants detected in tumour). The known *TP53* hotspot mutations, such as R248W, R175H and R273C, were among the mutations identified (see Table 4). The cases were categorised as follows: i) a group showing the LOF mutations or the above-mentioned hotspots (n = 9), ii) a *TP53* wild-type group (n = 5) and iii) a group consisting of unclassified *TP53* mutations (n = 17). The LOF and hotspot mutations were more commonly seen in the FIGO stage IV tumours (p = 0.003) and a low differentiation grade (p = 0.024).

**Table 4:** Overview over mutations detected in our cohort.

Mutation	AA-change	Responder to Carboplatin n (%)	Non-Responder to carboplatin n (%)
7577545 T > C	M246V^a^	1 (4.8%)	0
7577579 G > C	R248W^a*^	1 (4.8%)	1 (7.1%)
7578406 C > T	R175H^a*^	0	1 (7.1%)
7574002CG > C	FrameshiftR342*	1 (4.8%)	0
7578271 T > C	H193R^a^	1 (4.8%)	1 (7.1%)
7578190 T > C	Y220C^a^	0	1 (7.1%)
7577141 G > T	G266V^a^	1 (4.8%)	0
7577536 T > A	R249W^a^	1 (4.8%)	0
7577121 G > A	R273C^a*^	0	1 (7.1%)
7577106 G > A	P278S^a^	1 (4.8%)	0
7577570 C > T	M237I^a^	1 (4.8%)	0
7577548 C > T	G245S^a^	1 (4.8%)	1 (7.1%)
7577577 T > C	N235S^a^	0	1 (7.1%)
7577509 C > A	E258Xnonsense^a*^	0	1 (7.1%)
7578535 T > C	K132R^a^	1 (4.8%)	0
7572990CT > C	FrameshiftK373*	0	1 (7.1%)
7574017 C > A	R337L^a^	1 (4.8%)	0
7578527 A > C	C135G^a^	1 (4.8%)	0
7578210 T > C	R213R	1 (4.8%)	0
7579472 G > C	P72R^a^	2 (9.5%)	3 (21.4%)
7578271 T > A	H193L^a^	1 (4.8%)	0
7577556 C > G	C242S^a^	1 (4.8%)	0
7577121GCAC > G	DeletionR273^*^	0	1 (7.1%)
7577122 C > G	V272V^a^	0	1 (7.1%)
7578529 A > C	F134C^a^	1 (4.8%)	0
7578442 T > G	Y163S^a^	1 (4.8%)	0
7578442 T > A	K120Xnonsense^*^	1 (4.8%)	0

^a^Mutation has been reported earlier in the IARC archive^39^; *defined as hotspot or LOF mutation. Abbrevations: AA amino acid.

The possible correlation of the *TP53* mutation status with PFS as a surrogate for response to chemotherapy was tested. The patients carrying tumours with the three groups of mutations showed a significantly different response to chemotherapy (p = 0.029), with the unclassified mutations revealing the longest PFS when compared to the wild-type and the LOF/hotspot patients. Similar results were found with the univariate survival analysis (logrank p = 0.002; Fig. 4C). When introducing a combined biomarker panel including the *TP53* mutation classes and levels of p53 isoforms into a Kaplan-Meier univariate model, we did not observe an enhanced discrimination between the patient groups (data not shown).

## Discussion

The ability to predict response to first-line chemotherapy in HGSOC is currently limited. Better biomarkers are needed to optimise the therapeutic regimen for the patients. There are a limited number of studies assessing the prognostic relevance of the p53 network, including the aberrant expression of p53 isoforms reported in various human malignancies^13–17,23–26,29,30^. By quantification of the mRNA levels of selected p53 isoforms in HGSOC tumour tissues, from patients carefully selected based on chemotherapy responses, we showed that the overexpression of the total Δ133p53 isoforms was associated with prolonged OS and PFS in multivariate survival analyses. However, the p53 isoform levels were not found to be significantly differentially distributed when the chemoresistant and chemosensitive cohorts were compared. These results are in line with a previous study that identified the Δ133p53 isoforms (Δ133p53α,β,γ) as favourable prognostic factors in *TP53* mutated, advanced serous ovarian cancers^24^. In contrast, Δ133p53β has been shown to promote invasion and cancer cell stemness in breast cancer cell lines and has been associated with impaired survival in patients with breast carcinomas^16,17^. The exact roles of Δ133p53 isoforms and the means by which they become tissue-specific therefore remain to be elucidated. Furthermore, the higher relative expression of p53β to total p53 was associated with FIGO stage IIIC; thus, it could be hypothesised that p53β plays a role in the metastatic potential. The p53β isoform is missing the transactivation and tetramerization domains that seem necessary for enabling the transcriptional competence of p53^31^, and the higher expression has been associated with tumour traits and favourable survival in other cancer types^15,29^.

The present data also revealed that the mRNA level of Δ133p53 was much higher than the levels of p53β and p53γ in most patients. This is in line with previous findings for uterine serous carcinomas^26^. While the p53β and p53γ isoform may still be important, one may speculate that the low levels of these mRNAs compared to the total levels of p53 mRNA may limit their prognostic influence. We found expression levels of p53 isoforms to be highly correlated with each other. One possible explanation for this relationship is the presence of simultaneous alterations on both the amino- and carboxy-terminus of the protein. However, the isoform expression was not directly proportional to the total levels of p53 mRNA. It is therefore likely that isoforms are not merely a by-product of general p53 transcription but rather have their own biological importance. This controlled side-production of isoforms by the splice machinery may reduce the wild-type transcript effect. Additionally, some of the cases analysed maybe possess a more general, inherent splice chaos.

The *TP53* mutation status had predictive significance when the *TP53* mutations were categorised into (i) a group with LOF mutations and common hotspots (R273, R248, R175) that was compared with the survival of patients bearing cancers (ii) with the *TP53* wild-type tumours or (iii) other, unclassified *TP53* defects. There are conflicting reports on the predictive value of different *TP53* mutation types in HGSOC^6,7,32^. Similar to the findings in this study, other studies^33^ have also found the PFS in patients with *TP5*3 wild-type tumours inferior to that of patients with non-canonical *TP53* mutations. Because *TP53* mutations are an almost universal observation in HGSOC^4^, the observed wild-type status could be questioned. It is possible that *TP53* is inactivated by alternative mechanisms in the patients with no detected *TP53* mutation.

A correlation was found between *TP53* LOF and hotspot mutations and the FIGO stage IV tumours. This is in line with previous studies revealing differential effects of the various *TP53* mutations. The *TP53* mutations seem to have a differential oncogenic capacity, depending on the mutation site and resulting downstream effect^5^. The univariate analyses of the combined biomarker panels for the *TP53* mutation classes and the expression of p53 isoforms did not enhance the significance of the prognostic information. This might have been the result of the small number of patients analysed in each group (n = 5–8; data not shown).

The clinical assessment of the *TP53* mutation status are regularly performed by immuno-histochemical staining^34^. This standard diagnostic approach is based on the accumulation of p53 protein in a majority of *TP53* mutated cases. This accumulation of p53 protein is seen as the result of an increased protein stability of mutant p53. Here, we found that the *TP53* mutated specimens also had higher levels of total p53 mRNA, and this might have contributed to the resulting protein accumulation.

Notably, we found five patients to be *TP53* wild-type. While, there is an ongoing debate whether TP53 mutations are ubiquitous in HGSOC, it is noteworthy that large sequencing efforts, such as TCGA, have also found a fraction of HGSOC to be *TP53* wild-type^33^. As such we believe our findings regarding mutational status, represent the true biology of our cases.

The small number of cases resulting from the rigorous exclusion of possible confounders must be regarded as limitation of this study. In addition, the expression of the total Δ40p53 isoform was not detected in the current experiments although this isoform has been detected in comparable series^24^. Although the current experimental setup enabled the quantitative detection of mRNAs containing the specific breakpoints for the different p53 isoforms, we were unable to reveal the combined breakpoints leading to alterations on both the carboxy-terminus and the amino-terminus (total ∆133p53 includes the pool of ∆133p53α, ∆133p53β and ∆133p53γ isoforms). Currently, such mRNAs can be detected only qualitatively and, at best, semi-quantitatively. It is possible that isoform subtypes may possess an even better prognostic impact in HGSOC and other cancer types.

The ability to predict a good response to first-line chemotherapy in HGSOC is a crucial milestone in the implementation of personalised medicine. The term ‘BRCAness’ was introduced to describe ovarian or breast tumours that share molecular features and drug sensitivity with *BRCA*-mutated tumours^35^. ‘BRCAness’ is associated with the response to PARP inhibitors, but no other molecular biomarkers play a role in the treatment stratification for HGSOC. The findings of this study point to the Δ133p53 isoform as a potential biomarker, pending further validation.

## Methods

### Patient characteristics

The original cohort of 493 individuals represents a consecutive collection of all of the patients diagnosed with and treated for EOC at Haukeland University Hospital, Bergen, Norway, during the period August 2001–June 2013. From this cohort, 40 patients with a good response to standard combination chemotherapy (‘responders’, defined as time to recurrence ≥17 months; n = 40) and 29 patients with a poor response to treatment (‘non-responders’, defined as progressive disease ≤6 months; n = 29) were selected to participate in the study. Advanced HGSOC was defined as FIGO stage IIIC or IV, high-grade (World Health Organization [WHO] Grade 2/3) serous histology and ovarian, primary peritoneal or fallopian origin. The patient demographic data and tumour traits, such as FIGO stage, as well as phenotypic characteristics, such as date of diagnosis and surgical treatment, level of complete cytoreduction and follow-up (at a mean of 41 months, range 2–144 months) were available for analysis (see Table 1).

All of the patients included in the study were treated with primary cytoreductive surgery and clinically staged according to the FIGO 2014 criteria^36^. Surgical debulking was followed by platinum-based chemotherapy. Patients who were offered different treatment protocols, such as neoadjuvant chemotherapy and anti-angiogenic treatment, and patients who had concomitant cancers were excluded to avoid confounders. PFS was defined as the time interval, in months, between the date of the termination of adjuvant platinum-containing chemotherapy to the date of recurrence or last follow-up. OS was classified as an outcome measure and defined as the time interval, in months, from the day of the primary surgery to the last follow-up date or death from disease. Two patients were excluded from this part of the analysis because of missing follow-up data.

The study was conducted in accordance with the Declaration of Helsinki and approved by the local ethical committee (Regional Committees for Medical and Health Research Ethics Vest [REK], University of Bergen, Norway, id: 2018/72). The biological material and phenotypic data that formed the basis of this project are part of the Bergen Gynecologic Cancer Biobank, Women’s Clinic, Haukeland University Hospital, Bergen, Norway (REK id: 2014/1907 and 2015/548). In accordance with the local ethics committee’s guidelines, written informed consent was prospectively obtained from all of the women before the collection of fresh frozen tumour tissue, blood samples and clinicopathologic parameters was initiated.

### Tumour tissue

After collection at the time of primary diagnosis, the tissue specimens were immediately frozen in liquid nitrogen, and the clinical data were annotated. If there were multiple eligible primary biopsies, the ovarian mass was the preferred tumour site for analysis. The tumour content of the fresh frozen specimens was assessed in ethanol-fixed and hematoxylin- and eosin- stained sections. While the minimum cut-off for inclusion was set at 50%, the tumour purity was more than 80% in a majority of the included tissue samples (n = 39). The histopathological analysis was performed at the Haukeland University Hospital Department of Pathology. The specimens were fixed in buffered formaldehyde, embedded in paraffin and further processed in the laboratory before standard histological sections were made. Pathologists trained in gynaecologic oncology performed the diagnostic assessments.

### Nucleic acid isolation and cDNA synthesis

DNA was isolated from the fresh frozen samples by tissue digestion at 65 °C in a lysis buffer containing NaCl, EDTA 0.5 M pH8.5, TrisM pH8, sodium dodecyl sulphate (SDS) 5%, proteinase K 20 mg/ml and H_2_O. Following overnight incubation, standard ethanol precipitation with sodium perchlorate and isopropanol was performed. DNA quantity was determined using a Qubit fluorometer (Thermo Fisher Scientific, Waltham, MA, USA). RNA was isolated using the RNeasy Mini Kit (Qiagen, Hilden, Germany) according to the manufacturer’s instructions. RNA quantification was performed using a NanoDrop M-1000 spectrophotometer (Thermo Fisher Scientific, Waltham, MA, USA) and Agilent 2100 Bioanalyzer (Agilent Technologies, Santa Clara, CA, USA). A total of 500 ng RNA was added to 20 μL reaction mix, and single-strand cDNA was synthesised using the Transcriptor Reverse Transcriptase system (Roche, Basel, Switzerland) according to the manufacturer’s protocol.

### Quantitative real-time PCR

Quantitative real-time PCRs (qPCR) were performed using specific primers and hydrolysis probes (see Table-S1) targeting total *TP53* or the different isoforms of *TP53*, as was previously described^26^. Importantly, this experimental setup enabled the quantitative detection of the mRNA production of p53 isoforms; however, it did not allow for discrimination between the full-length molecules and their respective isoforms exhibiting alterations on both the carboxy-terminus and the amino-terminus. This important limitation is caused by the limited amplicon length used for the real-time qPCR. The mRNAs of the detected and quantified *TP53* were the total p53, total Δ133p53, p53β and p53γ isoforms.

### *TP53* sequencing and mutation calling

The targeted massive parallel sequencing of the tumour DNA generated data on the *TP53* mutation status. The fragmentation of 1,000 ng dsDNA was achieved using the Covaris® M220 Focused-ultrasonicator™ (Covaris, Woburn, MA, USA). The library preparation was performed using the Agilent SureSelectXT reagent kit (Agilent Technologies, Santa Clara, CA, USA), and the individual samples were run on a MiSeq instrument (Illumina, San Diego, CA, USA). This design included +/−10 nucleotides at exon-intron borders, to cover potential splice site mutations. The *TP53* data was extracted from a sequencing effort applying baits targeting 360 genes as previously described in detail^37^. Preliminary mutation calling was performed using the MiSeq Reporter (MSR) software, and the raw mutation calling output was revised by the application of post-processing filters. All of the suspected *TP53* mutations were validated by the manual inspection of the sequencing reads using the Integrative Genomics Viewer^38^.

### Statistical analyses

The Shapiro-Wilk test was performed to assess the normality assumption. On the basis of the non-normal distributed expression levels of total p53, total Δ40p53, total Δ133p53, p53β and p53γ isoforms, we calculated the Spearman’s rank-order correlation for those variables. The Mann-Whitney U- and Kruskal-Wallis tests were applied to investigate the associations among the continuous variables (age and isoform expression levels) and the categorical variables (age grouped by median, disease stage, histological grade, presence of complete cytoreduction and chemoresistance as well as *TP53* mutation status). Fisher’s exact test and Pearson’s chi-squared test were used for comparisons of the categorical variables (patient age grouped by median, presence of complete cytoreduction, chemosensitivity and mutation status). The univariate survival analysis was performed by the Kaplan-Meier method, and subsets of patients (divided by median absolute or relative to total p53 expression of isoforms) were compared using the log-rank test. Multivariate survival analyses were performed using the Cox proportional-hazards regression model in a one-step fashion. Important clinical predictors of survival, such as age, FIGO stage and resection status, were added as categorical covariates. In the Cox proportional-hazards model, the absolute expression of the p53 isoforms and the isoform expression relative to the total p53 were tested as predictors. All of the *p*-values were reported as two-sided, and p-values < 0.05 were considered significant. The statistical analyses were performed using the SPSS 22.0 software package (SPSS Inc., Chicago, IL, United States of America).

## Supplementary information

Supplementary material

## Data Availability

The datasets generated during and/or analysed during the current study are available from the corresponding author on reasonable request.
