# Supplementary material for: Influence of p53 Isoform Expression on Survival in High-Grade Serous Ovarian Cancers
Source: Sci Rep. 2019 Mar 27;9:5244. doi: 10.1038/s41598-019-41706-z (PMC6437169; doi:10.1038/s41598-019-41706-z)
Supplement: Supplementary file 1 — Supplementary material [file 41598_2019_41706_MOESM1_ESM.pdf]

INFLUENCE OF p53 ISOFORM EXPRESSION ON SURVIVAL IN HIGH-GRADE SEROUS OVARIAN CANCERS

Katharina Bischof[1,2], Stian Knappskog[3,4], Sigrun M. Hjelle[1,7], Ingunn Stefansson[5,6], Kathrine Woie[2], Helga B. Salvesen[1,2]†, Bjorn T. Gjertsen[1,7], Line Bjorge[1,2]*

[1]Centre for Cancer Biomarkers CCBIO, Department of Clinical Science, University of Bergen, 5020 Bergen, Norway

[2]Department of Gynecology and Obstetrics, Haukeland University Hospital, 5021 Bergen, Norway

[3]Department of Oncology, Haukeland University Hospital, 5021 Bergen, Norway

[4]Section of Oncology, Department of Clinical Science, University of Bergen, 5020 Bergen, Norway

[5]Department of Pathology, Haukeland University Hospital, 5021 Bergen, Norway

[6] Centre for Cancer Biomarkers CCBIO, Department of Clinical Medicine, Section for Pathology, University of Bergen, 5020 Bergen, Norway

[7]Department of Internal Medicine, Haematology Section, Haukeland University Hospital, 5021 Bergen, Norway

Email addresses: katharina.bischof@uib.no, stian.knappskog@uib.no, sigrun.hjelle@uib.no; ingunn.stefansson@uib.no, kathrine.woie@helse-bergen.no, bjorn.gjertsen@uib.no, line.bjorge@uib.no

* **Corresponding author:** Line Bjørge, Department of Gynecology and Obstetrics, Haukeland University Hospital, N-5021 Bergen, Norway. E-mail address: line.bjorge@uib.no, Telephone number: +4755974200, Fax number: +4755974968

Figure-S1: Kaplan-Meier survival plots comparing OS of patients with tumours that show higher than median expression of isoforms vs. lower than median levels for **A.** p53β mRNA absolute **B.** p53β mRNA relative to total p53 **C.** p53γ mRNA absolute **D.** p53γ mRNA relative to total p53.

Figure-S2: Kaplan-Meier survival plots comparing PFS for patients with tumours that show higher than median expression of isoforms vs. lower than median levels for **A.** p53β mRNA absolute **B.** p53β mRNA relative to total p53 **C.** p53γ mRNA absolute **D.** p53γ mRNA relative to total p53.

Figure-S1:

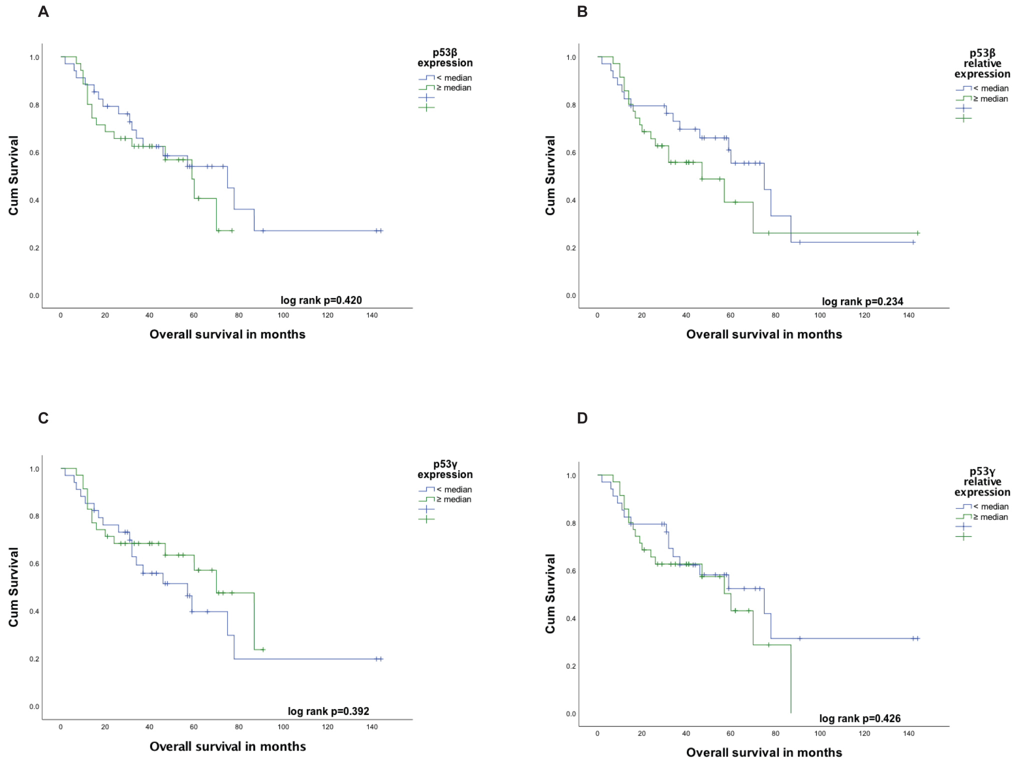

Figure-S2:

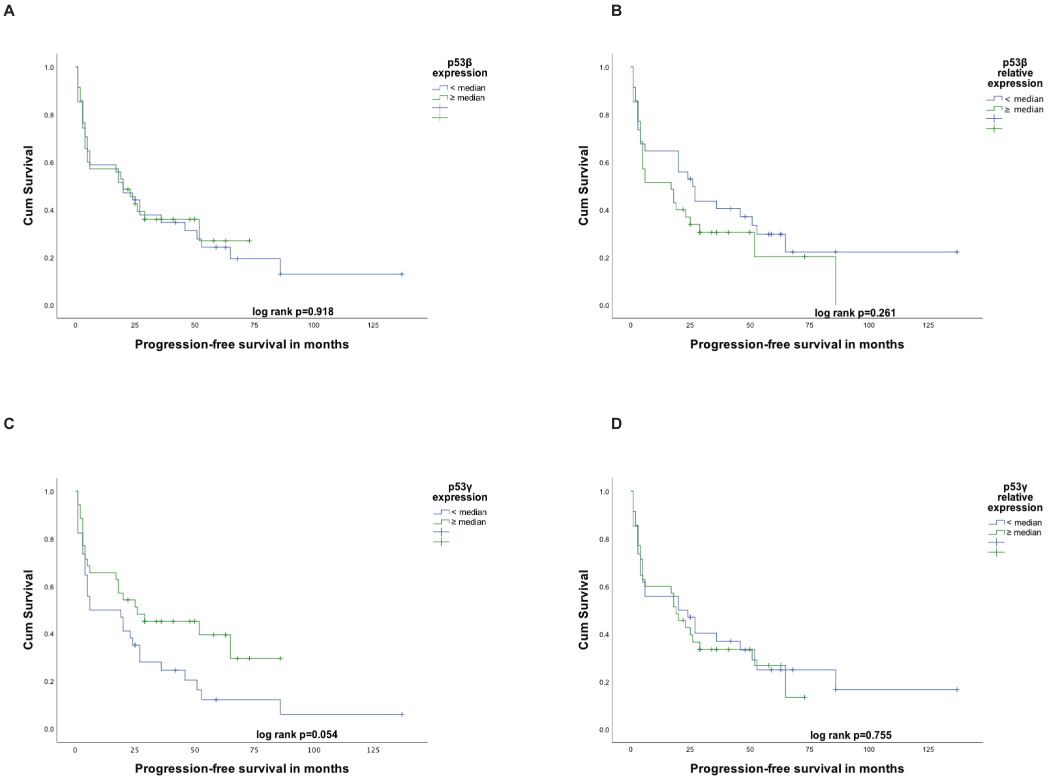
A
p53β expression
< median
≥ median
Cum Survival
log rank p=0.918
Progression-free survival in months
B
p53β relative expression
< median
≥ median
Cum Survival
log rank p=0.261
Progression-free survival in months
C
p53γ expression
< median
≥ median
Cum Survival
log rank p=0.054
Progression-free survival in months
D
p53γ relative expression
< median
≥ median
Cum Survival
log rank p=0.755
Progression-free survival in months

Table S1 - Primers and probes for qPCR.

Primer / probe	Sequence
p53 total	
p53 total F	GAAGAGAATCTCCGCAAGAAAGG
p53 total R	TCCATCCAGTGGTTTCTTCTTTG
p53 total probe	6FAM-AGCACTAAGCGAGCACTGCCCAACA-BBQ
Δ40p53	
Δ40p53	CAGACCTATGGAAACTGTGAGTGG
Δ40p53	TCAGGAAGTCTGAAAGACAAGAGCAG
Δ40p53	6FAM-CCTAGCAGAGACCTGTGGGAAGCGAA-BBQ
Δ133p53	
Δ133p53 F	ACTCTGTCTCCTTCCTCTTCCTACAG
Δ133p53 R	GTGTGGAATCAACCCACAGCT
Δ133p53 probe	6FAM-TCCCCTGCCCTCAACAAGATGTTTTGCC-BBQ
p53β	
p53β F	AACCACTGGATGGAGAATATTTCAC
p53β R	TCATAGAACCATTTTCATGCTCTCTT
p53β probe	6FAM-CAGGACCAGACCAGCTTTCAAAAAGAAAATTGTT-BBQ
p53δ	
p53δ F	AACCACTGGATGGAGAATATTTCAC
p53δ R	TCAACTTACGACGAGTTTATCAGGAA
p53δ probe	6FAM-TTCAGATGCTACTTGACTTACGATGG-BBQ
RPLP2	
RPLP2 F	GACCGGCTCAACAAGGTTAT
RPLP2 R	CCCCACCAGCAGGTACAC
RPLP2 probe	Cy5-AGCTGAATGGAAAAAACATTGAAGACGTC-BBQ

Table S2 - Prognostic and predictive impact of the p53 splice variant Δ133p53 for 69 women with HGSOC in multivariate Cox regression analysis.

	**Progression free survival**		**Overall survival**	
	**HR (95% CI)**	**P-value**	**HR (95% CI)**	**P-value**
**Age at diagnosis** in years	0.996 (0.959-1.035)	0.838	0.973 (0.930-1.018)	0.236
**Tumor stage** IIIC vs. IV	2.237 (1.028-4.886)	0.042	3.056 (1.193-7.830)	0.020
**Tumor grade** II vs. III	0.856 (0.415-1.763)	0.997	1.002 (0.397-2.530)	0.680
**Residual disease** 0 vs. >0	2.079 (0.918-4.710)	0.079	3.751 (1.035-13.588)	0.044
**Δ133p53** High vs. Low			0.585 (0.267-1.279)	0.179
**Δ133p53ratio** High vs. Low	0.705 (0.394-1.264)	0.241		
